# Household finished flooring and soil-transmitted helminth and *Giardia* infections among children in rural Bangladesh and Kenya: a prospective cohort study

**DOI:** 10.1016/S2214-109X(20)30523-4

**Published:** 2021-02-16

**Authors:** Jade Benjamin-Chung, Yoshika S Crider, Andrew Mertens, Ayse Ercumen, Amy J Pickering, Audrie Lin, Lauren Steinbaum, Jenna Swarthout, Mahbubur Rahman, Sarker M Parvez, Rashidul Haque, Sammy M Njenga, Jimmy Kihara, Clair Null, Stephen P Luby, John M Colford, Benjamin F Arnold

**Affiliations:** a Division of Epidemiology & Biostatistics, University of California, Berkeley, Berkeley, CA, USA; b Energy & Resources Group, University of California, Berkeley, Berkeley, CA, USA; c Department of Forestry and Environmental Resources, North Carolina State University, Raleigh, NC, USA; d Civil and Environmental Engineering, Tufts University, Medford, MA, USA; e Exponent, Sacramento, CA, USA; f International Centre for Diarrhoeal Disease Research, Dhaka, Bangladesh; g Eastern and Southern Africa Centre of International Parasite Control, Kenya Medical Research Institute, Nairobi, Kenya; h Center for International Policy Research and Evaluation, Mathematica Policy Research, Washington, DC, USA; i Division of Infectious Diseases and Geographic Medicine, Stanford University, Stanford, CA, USA; j Francis I Proctor Foundation, University of California, San Francisco, CA, USA; k Department of Ophthalmology, University of California, San Francisco, CA, USA

## Abstract

**Background:**

Soil-transmitted helminths and *Giardia duodenalis* are responsible for a large burden of disease globally. In low-resource settings, household finished floors (eg, concrete floors) might reduce transmission of soil-transmitted helminths and *G duodenalis*.

**Methods:**

In a prospective cohort of children nested within two randomised trials in rural Bangladesh and Kenya, we estimated associations between household finished flooring and soil-transmitted helminths and *G duodenalis* prevalence. In 2015–16, we collected stool samples from children aged 2–16 years in rural Bangladesh and Kenya. We detected soil-transmitted helminth infection using quantitative PCR (qPCR; Bangladesh n=2800; Kenya n=3094), and *G duodenalis* using qPCR in Bangladesh (n=6894) and ELISA in Kenya (n=8899). We estimated adjusted prevalence ratios (aPRs) using log-linear models adjusted for potential confounders.

**Findings:**

7187 (92·2%) of 7795 children in Bangladesh and 9077 (93·7%) of 9686 children in Kenya provided stool specimens that were analysed by qPCR. At enrolment, 691 (10%) households in Bangladesh and 471 (5%) households in Kenya had finished floors. In both countries, household finished flooring was associated with lower *Ascaris lumbricoides* prevalence (Bangladesh aPR 0·33, 95% CI 0·14–0·78; Kenya 0·62, 0·39–0·98) and any soil-transmitted helminths (Bangladesh 0·73, 0·52–1·01; Kenya 0·57, 0·37–0·88). Household finished floors were also associated with lower *Necator americanus* prevalence in Bangladesh (0·52, 0·29–0·94) and *G duodenalis* prevalence in both countries (Bangladesh 0·78, 0·64–0·95; Kenya 0·82, 0·70–0·97).

**Interpretation:**

In low-resource settings, living in households with finished floors over a 2-year period was associated with lower prevalence of *G duodenalis* and some soil-transmitted helminths in children.

**Funding:**

Bill & Melinda Gates Foundation and Task Force for Global Health.

## Introduction

Soil-transmitted helminths (eg, *Ascaris lumbricoides*, hookworm, and *Trichuris trichiura*) and *Giardia duodenalis* are estimated to infect over 1 billion people globally.^1^, ^2^ Infections can result in anaemia, malnutrition, and deficits in physical and cognitive development in children.^3^ Mass preventive chemotherapy is the primary control strategy for soil-transmitted helminths, but its effectiveness is limited by frequent reinfection after deworming, low deworming efficacy against *T trichiura*, and potential drug resistance.^1^, ^4^ Improved water, sanitation, and hygiene (WASH) interventions might also reduce transmission because soil-transmitted helminths and *G duodenalis* are transmitted through oral ingestion or dermal contact (in the case of hookworm) with faecally contaminated soil, food, water, or fomites*.*^3^, ^5^ Trials of household WASH interventions reported moderate reductions in prevalence of soil-transmitted helminths and *G duodenalis*, but effect sizes varied between countries and interventions.^5^, ^6^, ^7^

Improvements in poor quality housing are associated with improved health.^8^ In particular, installation of finished flooring (eg, wood, cement, or tile) might interrupt soil-transmitted helminths and *G duodenalis* transmission and does not require sustained behaviour change or extensive maintenance typical of many WASH interventions. Finished flooring might decrease transmission of soil-transmitted helminths and *G duodenalis* by reducing faecal contamination of hands, food, and water. Because soil-transmitted helminth ova survive best under moist conditions, impermeable finished flooring might reduce environmental survival and interrupt the lifecycle of soil-transmitted helminths, which requires a soil stage for soil-transmitted helminth ova to become infective.^3^ Studies in rural Kenya and urban Brazil detected soil-transmitted helminth ova at similar concentrations inside households and near latrines.^9^, ^10^ In low-resource settings, young children frequently ingest soil in households with dirt floors^11^ and detection of soil-transmitted helminths larvae in soil near children's play areas is associated with higher soil-transmitted helminth infection among children.^12^ Thus, there is biological plausibility for household finished flooring to reduce environmentally mediated enteric parasite transmission.

**Research in context**

**Evidence before this study**

We searched for primary studies and systematic reviews that investigated the association between household flooring and enteric infection or diarrhoea in Scopus using (TITLE-ABS-KEY((“household flooring” OR “dirt floors” OR “cement floors” OR “concrete floors”) AND (diarrhea* OR diarrhoea* OR parasite OR “soil-transmitted helminth” OR giardia OR enteric OR protozoa*))). We included all publications until May 15, 2020. We restricted results to studies in English in medicine, immunology or microbiology, agricultural and biological sciences, and environmental science. Our search yielded 63 studies. Earlier studies in both rural and urban settings reported associations between living in a household with a soil floor and infection with hookworm, *Ascaris lumbricoides*, *Strongyloides stercoralis*, *Giardia duodenalis*, cholera, other enteric parasite infections, and diarrhoea. With the exception of one study, which used a cohort design, all earlier studies used cross-sectional designs. One systematic review of housing improvements reported improvements in child and adult health.

**Added value of this study**

We investigated the association between household finished flooring and soil-transmitted helminth and *G duodenalis* infection in rural Bangladesh and Kenya. To our knowledge, ours is the first study to investigate this topic in a cohort of children born into households with different flooring conditions. We found that household finished flooring was associated with significantly lower prevalence of *A lumbricoides* and *G duodenalis* in both countries, and lower prevalence of *Necator americanus* in Bangladesh. This study improves on previous studies by analysing prospectively collected data from thousands of children in cohorts in Bangladesh and Kenya— a subset of which were followed since birth. In addition, this study used rigorous molecular diagnostic methods to detect infections that might be missed by more common copromicroscopic methods.

**Implications of all the available evidence**

Multiple observational studies, including ours with a prospective design, have reported large protective associations between household finished flooring and soil-transmitted helminth and enteric protozoan infection. A limitation of existing studies is that associations might be biased by unmeasured confounding, because flooring type is closely linked with socioeconomic status. Our results motivate future randomised trials of household finished flooring to measure its effect on enteric parasite infections.

Cross-sectional studies in urban and rural settings have reported associations between living in a household with a soil floor and higher risk of parasitic infections, including hookworm,^13^ *A lumbricoides,*^12^, ^14^, ^15^ *Strongyloides stercoralis,*^16^ and *G duodenalis*.^14^, ^17^ A retrospective, matched-impact evaluation^18^ of a government programme in urban Mexico found that replacing household dirt floors with concrete floors reduced diarrhoea and intestinal parasite infection in children.

We report the findings of a prospective cohort study of children nested within two cluster-randomised trials done in rural Bangladesh and Kenya. We investigated whether young children living in homes with finished floors (wood, tile, concrete) had lower prevalence and intensity of soil-transmitted helminths and *G duodenalis* infection 2 years after enrolment compared with children living in homes with unfinished (earthen) floors.

## Methods

### Study design

We did a prospective, observational analysis of children enrolled in the WASH benefits cluster-randomised trials in rural Bangladesh and Kenya. The trials delivered individual and combined water, sanitation, handwashing, and nutrition interventions to clusters of households to estimate effects on child growth, diarrhoea, and enteric infection 2 years after enrolment. Study groups comprised chlorinated drinking water (W); upgraded latrines (S); handwashing with soap (H); combined W, S, and H; breastfeeding promotion and lipid-based nutrient supplements (N); combined W, S, H, and N; and a control group containing twice the number of clusters in each of the other treatment groups.^19^, ^20^ In this analysis, we pooled across the study groups in each trial. The Bangladesh trial was done in rural villages in Gazipur, Kishoreganj, Mymensingh, and Tangail districts; and the Kenya trial was done in rural villages in western Kenya in Bungoma, Kakamega, and Vihiga counties. Trial populations were selected to be broadly representative of settings in rural, central Bangladesh, and western Kenya with high burdens of child growth failure and diarrhoea.^19^, ^20^ Additional trial details have been reported elsewhere.^5^, ^6^, ^7^, ^19^, ^20^

### Participants

Both trials defined birth cohorts by enrolling pregnant women and following their livebirths until approximately age 2 years. Children aged 18–27 months were also enrolled if present in the household. Enrolment occurred from May 31, 2012, to July 7, 2013, in Bangladesh and from Nov 27, 2012, to May 21, 2014, in Kenya. At approximately 2 years follow-up, the trials enrolled additional school-aged (3–16 years) children in the same households or compounds as children in the birth cohort. In Bangladesh, the trial enrolled up to two other children aged 3–12 years per compound. In Kenya, the trial enrolled one school-aged child per household by selecting the youngest available child aged 3–16 years. These additional enrolments occurred at the time of stool sample collection (May, 2015–May, 2016 in Bangladesh and January, 2015–July, 2016 in Kenya).

### Exposures

Field staff observed household flooring material at enrolment and 2-year follow-up and recorded each household's most common flooring material. We classified household floors observed at enrolment as finished if the most common floor material was wood, tile, or concrete, and as unfinished if the floor material was entirely made of soil or if it was mostly soil with small areas of wood, tile, or concrete.

### Outcomes

Prespecified, primary outcomes were prevalence of any soil-transmitted helminth infection measured by quantitative PCR (qPCR), and prevalence of *G duodenalis* infection measured by qPCR in Bangladesh and ELISA in Kenya. Secondary outcomes were species-specific prevalence and mean cycle quantification (Cq) values, geometric mean eggs per gram (epg), and moderate or heavy infection intensity measured by the Kato-Katz method.^21^, ^22^ Kato-Katz is a copromicroscopic diagnostic method that is frequently used to detect soil-transmitted helminths; it is inexpensive and feasible to perform in low-resource settings.

Studies tested for soil-transmitted helminths with multiparallel qPCR in a subsample of study groups (control, S, W, and combined W, S, and H in Bangladesh; control, combined W, S, and H, and combined W, S, H, and N in Kenya), and with double-slide Kato-Katz in all study groups. The primary analysis focused on qPCR-measured outcomes because it has higher sensitivity and specificity than Kato-Katz in settings with predominantly low infection intensity and because of potential misclassification of *A lumbricoides* using Kato-Katz in the Bangladesh trial.^23^, ^24^ We categorised soil-transmitted helminth infection intensity, using Kato-Katz based on WHO categories, as low, moderate, or heavy for hookworm (1–4999, 5000–49 999, or ≥50 000 epg, respectively), *A lumbricoides* (1–4999, 5000–49 999, or ≥50 000 epg, respectively), and *T trichiura* (1–999, 1000–9999, or ≥10 000 epg, respectively). Additional details on outcome measurement are available elsewhere (appendix pp 1–2).^5^, ^6^, ^7^, ^23^

### Statistical analysis

We estimated the minimum detectable effect assuming 80% statistical power, 5% type I error, and each country's sample size. We calculated infection prevalence of soil-transmitted helminths (36% Bangladesh, 29% Kenya) and *G duodenalis* (33% Bangladesh, 39% Kenya), and cluster-level design effects for soil-transmitted helminths (1·9 in Bangladesh, 1·7 in Kenya) and *G duodenalis* (1·5 in Bangladesh, 1·2 in Kenya). Minimum detectable prevalence ratios for any soil-transmitted helminths were 0·67 in Bangladesh and 0·58 in Kenya; for *G duodenalis*, they were 0·60 in Bangladesh and 0·82 in Kenya.

We registered a pre-analysis plan. We did analyses separately by country. We estimated prevalence ratios and prevalence differences using log-linear modified Poisson models.^25^ We estimated the relative difference in Cq values (the ratio of the arithmetic mean of Cq values –1) among children who were infected using linear models and used the delta method to obtain SEs. We also estimated associations using double-robust, targeted maximum likelihood estimation (TMLE) to optimise bias-variance tradeoff.^26^ Adjusted models controlled for each of the following prespecified covariates associated with the outcome (likelihood ratio test p values <0·20): month of sample collection, child age, child sex, child birth order (Bangladesh only), mother's age, mother's educational attainment (no education, primary, or secondary), mother's height (cm), number of individuals in the household aged 18 years or younger, number of individuals in the compound, food insecurity (using the Household Food Insecurity Access Scale^27^), household electricity, and household assets (eg, table or television ownership). Flooring status was well balanced across treatment groups among households that contributed parasite measurements, but we included study group as a covariate to adjust for any effects of WASH and nutritional interventions (improved sanitation, water quality, handwashing, or nutrition).^5^, ^6^, ^7^ We did not adjust for categorical covariates with prevalence of less than 5%. Robust SEs were clustered at the village cluster level.^25^ We assessed effect modification (prespecified) by birth cohort enrolment (*vs* older children), improved latrine access at enrolment, and caregiver-reported deworming in the past 6 months.

To diagnose and prevent positivity violations (a lack of experimentation in the exposure in a covariate stratum), we used an ensemble machine learning algorithm^28^ to estimate the predicted probability of having a finished floor adjusting for measured covariates. As a sensitivity analysis, we excluded observations with extreme predicted probabilities of having finished flooring (within 1% of the minimum and maximum predicted probability in each country). A small number of households changed flooring status between enrolment and 2-year follow-up. We did sensitivity analyses that excluded households that changed status and reclassified households that improved their flooring during the study as finished floor households.

To assess possible influence of bias from unmeasured confounding, we estimated E values, which quantify the relative risk that an unmeasured confounder would need to have with the exposure and outcome for bias to explain the observed association.^29^ Large E values (eg, >2–3-times increase in risk of the exposure and outcome) indicate that unmeasured confounding would be very unlikely to explain an observed association, whereas small E values (eg, 1**·**1-times increase) indicated that unmeasured confounding could potentially explain the association.

This study was done using publicly available, deidentified data. Full replication files are available online. The original trial protocols were approved by the committee for the protection of human subjects at the University of California, Berkeley (2011-09-3652, 2011-09-3654), the institutional review board at Stanford University (23310, 25863), the scientific and ethics review unit at the Kenya Medical Research Institute (SSC-2271), and the ethical review committee at The International Centre for Diarrhoeal Disease Research, Bangladesh (PR-11063).

### Role of the funding source

The funders of the study approved the study design, but had no role in data collection, data analysis, data interpretation, or writing of the report. The corresponding author had full access to all data in the study and had final responsibility for the decision to submit for publication.

## Results

In Bangladesh, from 7795 children enrolled at 2 year follow-up, 7187 (92·2%) provided stool specimens (appendix p 3). In Kenya, from 9686 children enrolled at 2 year follow-up, 9077 (93·7%) provided stool specimens. Kato-Katz was used to detect soil-transmitted helminths in 100% of stool specimens in Bangladesh and 99·6% of specimens in Kenya. qPCR was used to detect soil-transmitted helminths in a subsample in each country (Bangladesh n=2800; Kenya n=3093). Sufficient stool for an additional aliquot was available to detect *G duodenalis* in 6894 (88·4%) specimens in Bangladesh and 8899 (91·8%) specimens in Kenya. The median child age was 2·7 years (IQR 2·5–7·1) in Bangladesh and 2·2 years (2·0–4·7) in Kenya. 4564 (64·6%) children in Bangladesh and 3815 (42·0%) children in Kenya were reported to have consumed deworming medication in the previous 6 months.

The prevalence of household finished flooring at enrolment was 9·6% in Bangladesh and 5·2% in Kenya. Fewer than 5% of households changed household flooring status between baseline and 2 year follow-up. In each country, some maternal and household characteristics differed among households with and without household finished flooring at enrolment (table). In Bangladesh, the proportion of mothers with at least some secondary education was 82·5% versus 48·3% in households with versus without finished floors. In Bangladesh and Kenya, households with finished floors were more likely to have electricity, improved wall and roof materials, and a television, motorcycle, and mobile telephone compared with households without finished floors.

**Table:** Characteristics at enrolment by household finished flooring status

	**Bangladesh**		**Kenya**	
	Finished floors	Unfinished floors	Finished floors	Unfinished floors
**Maternal characteristics**				
Mother's age, years	689, 24·2	6472, 24·3	467, 28·7	8531, 26·9
Mother's height, cm	687, 152·0	6475, 150·4	435, 161·8	8240, 160·1
At least some primary education	691, 13·5%	6496, 34·1%	471, 51·0%	8599, 78·8%
At least some secondary education	691, 82·5%	6496, 48·3%	471, 49·0%	8599, 21·1%
**Compound characteristics**				
Number of individuals living in compound ≤18 years	691, 1·7	6496, 1·7	471, 3·5	8597, 3·0
Total individuals living in compound	691, 10·0	6496, 11·8	471, 7·4	8599, 8·5
**Household characteristics**				
Food secure*	691, 91·3%	6496, 65·6%	471, 96·4%	8599, 88·5%
Has electricity	691, 88·7%	6496, 56·2%	471, 29·3%	8599, 6·5%
Has improved wall materials	691, 92·2%	6496, 71·1%	471, 70·5%	8599, 0·7%
Has improved roof material	691, 100·0%	6496, 98·2%	471, 97·7%	8599, 65·2%
Owns ≥1 television	691, 69·8%	6496, 25·0%	471, 41·4%	8599, 11·0%
Owns ≥1 bicycle	691, 35·0%	6496, 31·6%	471, 63·9%	8599, 54·2%
Owns ≥1 motorcycle	691, 23·4%	6496, 4·4%	471, 25·1%	8599, 8·5%
Owns ≥1 mobile telephone	691, 98·6%	6496, 83·9%	471, 94·7%	8599, 80·4%
**Child characteristics**				
Child age, years	691, 4·7	6496, 4·8	449, 3·7	8279, 3·6
Female	691, 50.9%	6496, 51·0%	471, 45·0%	8599, 48·6%
Male	691, 49·1%	6496, 49·0%	471, 51·0%	8599, 47·6%

Data are n, mean or n, %.

* Assessed by the Household Food Insecurity Access Scale.

Using qPCR, prevalence of soil-transmitted helminth infection was 34·6% in Bangladesh and 28·3% in Kenya, and *G duodenalis* prevalence was 31·8% in Bangladesh and 38·7% in Kenya. Using Kato-Katz, the percentage of children with moderate or heavy infection intensity for *A lumbricoides* was 12% in Kenya, and less than 11·5% for hookworm and *T trichiura* in both countries. Comparing households with finished versus unfinished flooring, the prevalence of any soil-transmitted helminth infection measured using qPCR was 14·5% versus 36·7% in Bangladesh, respectively, and 14·5% versus 29·2% in Kenya, respectively (appendix p 8). Prevalence of soil-transmitted helminths was substantially lower when measured by Kato-Katz compared with qPCR (appendix p 9).

**Figure 1:**
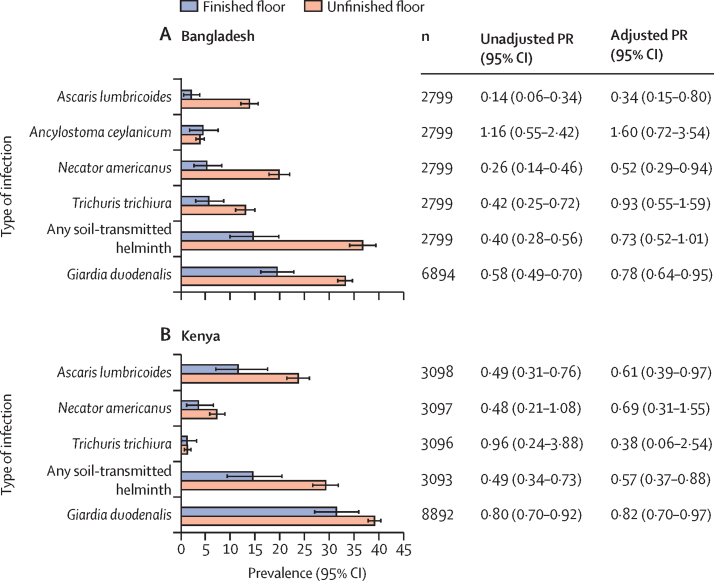
Soil-transmitted helminth and *Giardia duodenalis* prevalence and unadjusted and adjusted prevalence ratios comparing infection at 2 year follow-up by household flooring status at enrolment Finished floor includes majority wood, concrete, or tile household floors; unfinished floors were made of soil or earth. In the plots, error bars indicate 95% CIs. PRs compared the prevalence of infection at 2 year follow-up in children with improved household flooring at enrolment with those with unimproved household flooring at enrolment. Adjusted PRs control for potential confounders associated with the outcome. CIs were adjusted for clustering at the village level. *Ancylostoma duodenale* was only detected in one sample in each country, so it is excluded from this figure. PR=prevalence ratio.

Children living in households with finished flooring had a lower prevalence of *A lumbricoides*, *Necator americanus*, *T trichiura*, and any soil-transmitted helminth infection in Bangladesh, and *A lumbricoides* and any soil-transmitted helminth infection in Kenya (figure 1). After adjusting for potential confounders, household finished flooring was associated with lower prevalence of *A lumbricoides* in both countries (Bangladesh adjusted prevalence ratio [aPR] 0·34, 95% CI 0·15–0·80; Kenya 0·61, 0·39–0·97) and *N americanus* in Bangladesh (0·52, 0·29–0·94)*.* Household finished flooring was not associated with *Ancylostoma ceylanicum* and *T trichiura* prevalence in Bangladesh or *N americanus* and *T trichiura* in Kenya. For any soil-transmitted helminth prevalence, aPRs were 0·73 (0·51–1·01) in Bangladesh and 0·57 (0·37–0·88) in Kenya. For soil-transmitted helminths detected using Kato-Katz, aPRs were similar to those detected by qPCR, except that there was a statistically significant reduction in *T trichiura* infection in Bangladesh using Kato-Katz that was not observed when using qPCR (0·57, 0·33–0·96; appendix p 10). Household finished floors were not associated with moderate or heavy *A lumbricoides* infection in either country (Bangladesh 0·56, 0·24–1·31; Kenya 0·71, 0·45–1·10). In both countries, household finished flooring was associated with lower *G duodenalis* prevalence (Bangladesh 0·78, 0·64–0·95; Kenya 0·82, 0·70–0·97; figure 1).

**Figure 2:**
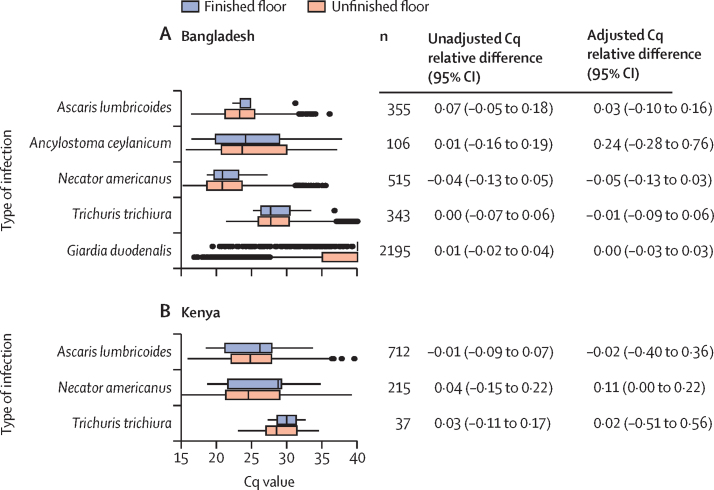
Soil-transmitted helminth and *Giardia duodenalis* Cq value and unadjusted and adjusted relative difference in Cq value among infected individuals at 2 year follow-up by household flooring status at enrolment Finished floor includes majority wood, concrete, or tile household floors; unfinished floors were made of soil or earth. Box plots show median (IQR), and whiskers show 1·5 times the IQR. Relative differences in Cq values were estimated as the ratio of the arithmetic mean Cq value at 2 year follow-up in children with improved household flooring at enrolment to those with unimproved household flooring at enrolment minus 1. Adjusted relative differences in Cq values control for potential confounders associated with the outcome. CIs were adjusted for clustering at the village level. *Ancylostoma duodenale* was only detected in one sample in each country, so it is excluded from this figure. Cq=cycle quantification.

Among children with infections detected by qPCR, Cq values were similar in children with versus without finished flooring (figure 2). Among children with soil-transmitted helminth infections detected by Kato-Katz, there were statistically significant adjusted faecal egg count differences for *T trichiura* in Bangladesh (–0·45, 95% CI –0·59 to –0·32; appendix pp 4, 11). Analyses using doubly robust TMLE produced slightly different point estimates, but overall were consistent with primary analyses using log-linear models (appendix pp 12–13).

There was no evidence of statistically significant effect modification on the multiplicative scale by child deworming consumption or improved latrine access at enrolment in either country (appendix p 5). Compared with older children, children in the birth cohort had a stronger protective association between finished flooring and *G duodenalis* prevalence in Bangladesh (birth cohort aPR 0·68, 95% CI 0·52–0·91; older children 0·86, 0·69–1·08; p_interaction_=0·02).

In a sensitivity analysis excluding observations with extreme predicted probabilities of having a finished floor (appendix p 6), in Kenya, household characteristics were very similar to the full set of observations (appendix p 14). In Bangladesh, household characteristics were similar in households with a finished floor, but among households without a finished floor there were lower levels of household electricity, food security, and other indicators of socioeconomic status after excluding observations with extreme predicted probabilities. Overall, aPRs were similar when excluding observations with extreme predicted probabilities (appendix p 15). Sensitivity analyses reclassifying household flooring status to account for the small number of households that changed status during follow-up produced similar results as the primary analysis (appendix p 7).

For outcomes with statistically significant associations in the primary analysis, E values ranged from 1·2 for *G duodenalis* to 5·3 for *A lumbricoides* in Bangladesh and from 1·4 for *G duodenalis* to 1·9 for *A lumbricoides* in Kenya (appendix p 16).

## Discussion

In this prospective, observational cohort, we found that household finished flooring was associated with lower soil-transmitted helminths and *G duodenalis* infection in young children. In Bangladesh, household finished flooring was associated with lower prevalence of *A lumbricoides*, *N americanus,* any soil-transmitted helminths, and *G duodenalis*, but not *A ceylanicum and T trichiura*. In Kenya, we found protective associations with *A lumbricoides*, any soil-transmitted helminths, and *G duodenalis*, but not *N americanus* or *T trichiura*. Among infected individuals, household finished flooring was not associated with a difference in Cq values, but was associated with lower mean epg using Kato-Katz for *T trichiura* in Bangladesh and *A lumbricoides* in Kenya. Results varied by species of soil-transmitted helminths and by country, but overall these results suggest that household finished floors hold promise as an environmental intervention to reduce soil-transmitted helminth and *G duodenalis* transmission among children in low-resource settings. Our study improves on earlier studies by prospectively measuring infections with molecular detection methods.^30^ In addition, the study populations included children born into households with different flooring material, allowing us to investigate associations between household flooring and infection in early life.

Our findings are consistent with two earlier studies that used observational designs to estimate the association between household finished flooring and enteric parasite infection. A cross-sectional study^15^ in Bangladesh reported significant protective associations of similar magnitude with *A lumbricoides* and hookworm prevalence and also found no association with *T trichiura* in children and women aged 15–49 years. A retrospective, matched intervention study^18^ of a concrete flooring programme in Mexico reported a 21% reduction in parasite count (species not reported) among households whose dirt floors were fully replaced by concrete floors.

The magnitude of protective associations for household flooring was similar to or greater than the typical effect size for WASH and preventive chemotherapy interventions. WASH interventions in the same study populations reduced soil-transmitted helminths prevalence by up to 33% (results varied by species and intervention)^5^, ^6^ and *G duodenalis* prevalence by up to 25% in Bangladesh^7^ (in Kenya, interventions did not reduce prevalence^5^). A meta-analysis of randomised and quasirandomised studies estimated that mass deworming for soil-transmitted helminths delivered for at least 6 months reduced soil-transmitted helminth prevalence by 77% for *A lumbricoides* and 33% for hookworm (there was no effect on *T trichiura*).^31^ In this study, household finished flooring was associated with 38–67% lower *A lumbricoides* prevalence and 18–22% lower *G duodenalis* prevalence in both countries and 48% lower *N americanus* prevalence in Bangladesh. In earlier studies, neither WASH interventions, preventive chemotherapy, nor household finished flooring were observed to be associated with lower *T trichiura* prevalence.^5^, ^6^, ^15^, ^31^ Here, when using Kato-Katz, we found a significant 43% lower *T trichiura* prevalence among children in households with finished floors.

Household finished floors have several advantages over other interventions to reduce soil-transmitted helminth and *G duodenalis* infection. Compared with WASH interventions, household finished floors require minimal sustained behaviour change and are easy to maintain. For control of soil-transmitted helminth infections, household finished flooring might complement preventive chemotherapy, which has high reinfection rates after treatment.^4^ Another potential benefit of flooring and other housing improvements is increased happiness and improved mental health.^8^, ^18^

Household socioeconomic status is probably a strong confounder of household flooring material and enteric parasite infection, so it is possible that residual, unmeasured confounding influenced our findings.^32^ We assessed the role of unmeasured confounding using E values,^29^ which suggested that for unmeasured confounding to fully explain our statistically significant findings, the relative-scale association between unmeasured confounders and our exposures and outcomes would need to be 1·19–5·28 in Bangladesh and 1·43–1·87 in Kenya. The effect of unmeasured confounding might be even stronger given that our exposure was relatively rare (10% of participants in Bangladesh, and 5% in Kenya). Future randomised studies of household flooring would be able to minimise unmeasured confounding to rigorously estimate the effect of household flooring on enteric parasite infection.

This study had additional limitations. First, we only measured outcomes in children, but household finished flooring might also reduce infection among adults. We expect that reductions would be largest among individuals who spend more time at home, namely young children, women, and older people. Second, some soil-transmitted helminth species were rare in our study populations (eg, *A ceylanicum* in Bangladesh, and hookworm and *T trichiura* in Kenya), so those estimates had low precision. Third, Cq values were not normalised across plates using standard curves, which could have resulted in more variable estimates, although we would not expect systematic bias. Finally, an earlier study found evidence of an interaction between household flooring and household sanitation for certain soil-transmitted helminths.^15^ We assessed interaction by baseline improved sanitation (appendix p 5), but subgroup analyses might have been underpowered.

Strengths of the study include use of qPCR to measure infection and prospective data collection. Repeating the analysis in two countries with differing ecologies and disease transmission dynamics should increase the study's generalisability to other rural, low-resource settings. More than half of children were enrolled at birth (53%), allowing us to measure the association between household flooring and newly acquired infections during the first years of life. Our finding that improved flooring was associated with *G duodenalis* more among preschool-aged (~2 years) children supports the biological plausibility of dirt floors as a source of exposure to faecal contamination in early life, because younger children are most likely to be exposed in the home.

Household finished flooring holds promise as a potential intervention to reduce enteric parasites such as soil-transmitted helminths and *G duodenalis* in low-resource settings. Compared to WASH interventions, which also target enteric parasite transmission pathways, a key potential advantage of household finished flooring is that it requires minimal maintenance and behaviour change. Our findings motivate future randomised studies to rigorously assess the effect of household finished flooring on infection.

For the **pre-analysis plan** see https://osf.io/mtf8x/

For **full replication files** see https://osf.io/dgkw5/

For **full replication files** see https://osf.io/dgkw5/

## Data sharing

This study was done using publicly available, deidentified data. Full replication files are available online.

## References

[1] Moser W, Schindler C, Keiser J (2017). Efficacy of recommended drugs against soil transmitted helminths: systematic review and network meta-analysis. BMJ.

[2] Pires SM, Fischer-Walker CL, Lanata CF (2015). Aetiology-specific estimates of the global and regional incidence and mortality of diarrhoeal diseases commonly transmitted through food. PLoS One.

[3] Brooker S, Clements AC, Bundy DA (2006). Global epidemiology, ecology and control of soil-transmitted helminth infections. Adv Parasitol.

[4] Jia T-W, Melville S, Utzinger J, King CH, Zhou X-N (2012). Soil-transmitted helminth reinfection after drug treatment: a systematic review and meta-analysis. PLoS Negl Trop Dis.

[5] Pickering AJ, Njenga SM, Steinbaum L (2019). Effects of single and integrated water, sanitation, handwashing, and nutrition interventions on child soil-transmitted helminth and *Giardia* infections: a cluster-randomized controlled trial in rural Kenya. PLoS Med.

[6] Ercumen A, Benjamin-Chung J, Arnold BF (2019). Effects of water, sanitation, handwashing and nutritional interventions on soil-transmitted helminth infections in young children: a cluster-randomized controlled trial in rural Bangladesh. PLoS Negl Trop Dis.

[7] Lin A, Ercumen A, Benjamin-Chung J (2018). Effects of water, sanitation, handwashing, and nutritional interventions on child enteric protozoan infections in rural Bangladesh: a cluster-randomized controlled trial. Clin Infect Dis.

[8] Thomson H, Thomas S, Sellstrom E, Petticrew M (2009). The health impacts of housing improvement: a systematic review of intervention studies from 1887 to 2007. Am J Public Health.

[9] Steinbaum L, Njenga SM, Kihara J (2016). Soil-transmitted helminth eggs are present in soil at multiple locations within households in rural Kenya. PLoS One.

[10] Schulz A, Kroeger A (1992). Soil contamination with *Ascaris lumbricoides* eggs as an indicator of environmental hygiene in urban areas of north-east Brazil. J Trop Med Hyg.

[11] Kwong LH, Ercumen A, Pickering AJ, Unicomb L, Davis J, Luby SP (2016). Hand- and object-mouthing of rural Bangladeshi children 3–18 months old. Int J Environ Res Public Health.

[12] Krause RJ, Koski KG, Pons E, Sandoval N, Sinisterra O, Scott ME (2015). Ascaris and hookworm transmission in preschool children from rural Panama: role of yard environment, soil eggs/larvae and hygiene and play behaviours. Parasitology.

[13] Halliday KE, Oswald WE, McHaro C (2019). Community-level epidemiology of soil-transmitted helminths in the context of school-based deworming: baseline results of a cluster randomised trial on the coast of Kenya. PLoS Negl Trop Dis.

[14] Basualdo JA, Córdoba MA, de Luca MM (2007). Intestinal parasitoses and environmental factors in a rural population of Argentina, 2002–2003. Rev Inst Med Trop São Paulo.

[15] Benjamin-Chung J, Nazneen A, Halder AK (2015). The interaction of deworming, improved sanitation, and household flooring with soil-transmitted helminth infection in rural Bangladesh. PLoS Negl Trop Dis.

[16] Hall A, Conway DJ, Anwar KS, Rahman ML (1994). *Strongyloides stercoralis* in an urban slum community in Bangladesh: factors independently associated with infection. Trans R Soc Trop Med Hyg.

[17] Cociancic P, Torrusio SE, Zonta ML, Navone GT (2020). Risk factors for intestinal parasitoses among children and youth of Buenos Aires, Argentina. One Health.

[18] Cattaneo MD, Galiani S, Gertler PJ, Martinez S, Titiunik R (2009). Housing, health, and happiness. Am Econ J Econ Policy.

[19] Null C, Stewart C, Pickering A (2018). Effects of water quality, sanitation, handwashing and nutritional interventions on diarrhoea and child growth in rural Kenya: a cluster randomized trial. Lancet Glob Health.

[20] Luby S, Rahman M, Arnold B (2018). Effects of water quality, sanitation, handwashing and nutritional interventions on diarrhoea and child growth in rural Bangladesh: a cluster randomized trial. Lancet Glob Health.

[21] Kato K, Miura M (1954). Comparative examinations. Jpn J Parasitol.

[22] Katz N, Chaves A, Pellegrino J (1972). A simple device for quantitative stool thick-smear technique in *Schistosomiasis mansoni*. Rev Inst Med Trop São Paulo.

[23] Benjamin-Chung J, Pilotte N, Ercumen A (2020). Comparison of multi-parallel qPCR and double-slide Kato-Katz for detection of soil-transmitted helminth infection among children in rural Bangladesh. PLoS Negl Trop Dis.

[24] Easton AV, Oliveira RG, O'Connell EM (2016). Multi-parallel qPCR provides increased sensitivity and diagnostic breadth for gastrointestinal parasites of humans: field-based inferences on the impact of mass deworming. Parasit Vectors.

[25] Yelland LN, Salter AB, Ryan P (2011). Performance of the modified Poisson regression approach for estimating relative risks from clustered prospective data. Am J Epidemiol.

[26] van der Laan MJ, Rose S (2011). Targeted learning: causal inference for observational and experimental data.

[27] Ballard T, Coates J, Swindale A, Deitchler M (2011). Household hunger scale: indicator definition and measurement guide.

[28] van der Laan MJ, Polley EC, Hubbard AE (2007). Super learner. Stat Appl Genet Mol Biol.

[29] VanderWeele TJ, Ding P (2017). Sensitivity analysis in observational research: introducing the e-value. Ann Intern Med.

[30] O'Connell EM, Nutman TB (2016). Molecular diagnostics for soil-transmitted helminths. Am J Trop Med Hyg.

[31] Clarke NE, Clements ACA, Doi SA (2017). Differential effect of mass deworming and targeted deworming for soil-transmitted helminth control in children: a systematic review and meta-analysis. Lancet.

[32] Arnold BF, Null C, Luby SP, Colford JM (2018). Implications of WASH benefits trials for water and sanitation–Authors' reply. Lancet Glob Health.

