# Supplementary material for: Household finished flooring and soil-transmitted helminth and Giardia infections among children in rural Bangladesh and Kenya: a prospective cohort study
Source: Lancet Glob Health. 2021 Feb 16;9(3):e301–8. doi: 10.1016/S2214-109X(20)30523-4 (PMC7900607; doi:10.1016/S2214-109X(20)30523-4)
Supplement: Supplementary appendix [file mmc1.pdf]

THE LANCET
Global Health

Supplementary appendix

This appendix formed part of the original submission and has been peer reviewed. We post it as supplied by the authors.

Supplement to: Benjamin-Chung J, Crider YS, Mertens A, et al. Household finished flooring and soil-transmitted helminth and *Giardia* infections among children in rural Bangladesh and Kenya: a prospective cohort study. *Lancet Glob Health* 2021; **9:** e301–08.

Appendix to *Household finished flooring and soil-transmitted helminth and Giardia infections among children in rural Bangladesh and Kenya: a prospective cohort study*

Appendix 1. Outcome measurement

Stool collection and storage

Field staff provided primary caregivers of study participants with sterile containers. They returned the following morning to collect stool from the child's most recent defecation event. After stool collection, all study participants received a single dose of albendazole. Stool specimens were transported on ice to the field laboratory in each country, and 1 g of stool was archived in 1 ml of 100% ethanol. Stool as at -20°C until it was moved to a -80°C freezer.

Soil-transmitted helminth detection using Kato-Katz

On the day of stool collection, trained technicians performed double-slide Kato- Katz on fresh stool within 30 minutes of preparing each sample. Field staff enumerated *Ascaris lumbricoides*, hookworm, and *Trichuris trichiura* ova. For quality assurance, in Bangladesh 10% of slides were evaluated independently by two technicians, and 5% were evaluated by the same experienced parasitologist who conducted Kato-Katz training. In Kenya, 10% of slides were evaluated by an expert parasitologist.

Soil-transmitted helminth detection using Multi-parallel qPCR

Preserved stool samples from Bangladesh were shipped to Smith College in Northampton, MA, United States approximately 1–2 years after stool collection for qPCR analyses. Before shipment lab technicians evaporated from ethanol all samples in order to comply with shipping regulations. Samples were shipped on dry ice. Upon arrival at Smith College, samples were stored at -20°C until analysis. Preserved stool samples from Kenya were transported to the Eastern and Southern Africa Centre of International Parasite Control laboratory at the Kenya Medical Research Institute in Nairobi, Kenya, for analysis.

Lab technicians extracted DNA using the FastDNA Spin Kit for Soil (MP Biomedicals, Solon, OH) using a previously published modified version of the manufacturer's methodology.[1] Lab technicians used an internal amplification control (IAC) plasmid during the DNA extraction to ensure successful isolation and adequate DNA recovery. The mean and standard deviation of the mean quantitation cycle (Cq) value for IAC results was calculated from all samples. Lab technicians performed experimental qPCR reactions using multi-parallel assays that target non-coding repetitive sequences to detect *A. lumbricoides*[2], *T. trichiura*, *Necator americanus*, *Ancylostoma duodenale*[3] and *Ancylostoma ceylanicum*[4] (Bangladesh only). Lab technicians tested all samples in replicate reactions. We classified samples as positive if amplification occurred in both reactions with a Cq value of <40 and samples which produced Cq values ≥ 40 in both replicate reactions or failed to amplify in both replicate reactions as negative. A random subsample of stool samples from Kenya were shipped to Smith College for quality assurance.

G. duodenalis detection using qPCR (Bangladesh)

Lab technicians extracted DNA from 200 mg of stool using the QIAamp Fast DNA Stool Mini Kit (QIAGEN, Hilden, Germany). They eluted the DNA in 200 μl of ATE buffer (supplied with the QIAGEN kit). The protozoa were measured by multiplex real-time PCR using a previously published protocol.[5] Primers

[1]Mejia R, Vicuna Y, Broncano N, Sandoval C, Vaca M, Chico M, et al. A Novel, Multi-Parallel, Real-Time Polymerase Chain Reaction Approach for Eight Gastrointestinal Parasites Provides Improved Diagnostic Capabilities to Resource-Limited At-Risk Populations. *Am J Trop Med Hyg.* 2013; 88: 1041–1047. https://doi.org/10.4269/ajtmh.12-0726 PMID: 23509117

[2]Pilotte N, Maasch JRMA, Easton AV, Dahlstrom E, Nutman TB, Williams SA. Targeting a highly repeated germline DNA sequence for improved real-time PCR-based detection of Ascaris infection in human stool. *PLoS Negl Trop Dis. 2019*; 13: e0007593. https://doi.org/10.1371/journal.pntd.0007593 PMID: 31329586

[3]Pilotte N, Papaiakovou M, Grant JR, Bierwert LA, Llewellyn S, McCarthy JS, et al. Improved PCR- Based Detection of Soil Transmitted Helminth Infections Using a Next-Generation Sequencing Approach to Assay Design. *PLoS Negl Trop Dis.* 2016; 10: e0004578. https://doi.org/10.1371/journal. pntd.0004578 PMID: 27027771

[4]Papaiakovou M, Pilotte N, Grant JR, Traub RJ, Llewellyn S, McCarthy JS, et al. A novel, species-spe- cific, real-time PCR assay for the detection of the emerging zoonotic parasite Ancylostoma ceylanicum in human stool. *PLoS Negl Trop Dis.* 2017; 11: e0005734. https://doi.org/10.1371/journal.pntd.0005734 PMID: 28692668

[5]Haque R, Roy S, Siddique A, et al. Multiplex real-time PCR assay for detection of Entamoeba histolytica, Giardia intestinalis, and Cryptosporidium spp. *Am J Trop Med Hyg*, 2007; 76(4): 713-717.

and probes were purchased from Integrated DNA Technologies (Singapore). Amplification reactions were performed in a volume of 25 µL with 12.5 µL of QIAGEN Multiplex PCR Master Mix (QIAGEN, Hilden, Germany). Each reaction contained an additional 0.4 μM of each Gd-80F, Gd-127R primers and 0.04 μM of Gd-FT probes and 3 μl of the DNA sample. Samples were initial denatured for 15 minutes at 95°C, denatured at 95°C for 20 seconds for 40 cycles, and annealed at 60°C for 1 minute with fluorescence data collection. Lab technicians performed amplification and analysis on the CFX96 Real-Time System (BioRad, Hercules, CA). Each run included three positive controls and one negative control. We classified a sample as positive if its Ct was $<$40.

G. duodenalis detection using ELISA (Kenya)

Lab technicians at Kenya Medical Research Institute in Nairobi, Kenya, analyzed stool by monoclonal enzyme-linked immunosorbent assay (ELISA) (Giardia II, Alere International, Galway, Ireland) to detect *G. duodenalis* cysts. Samples were measured in duplicate; discrepant samples were rerun.

Appendix Figure 1. Enrollment flow chart

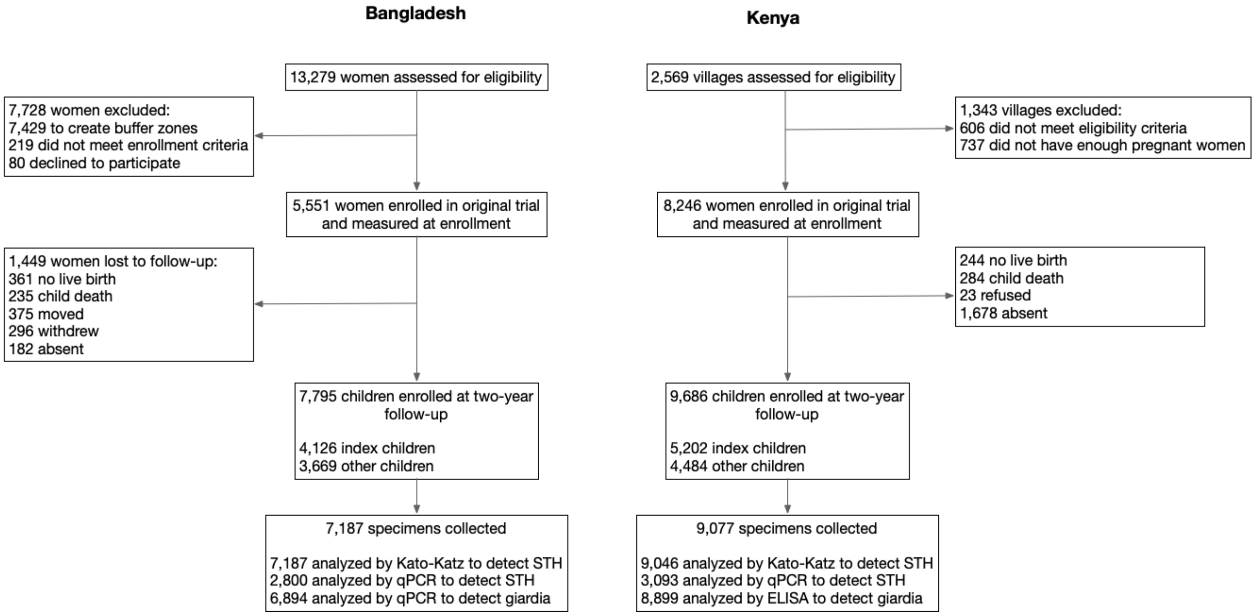

Appendix Figure 2. Eggs per gram among individuals with STH infections detected by Kato-Katz at two-year follow-up by household flooring status at enrollment

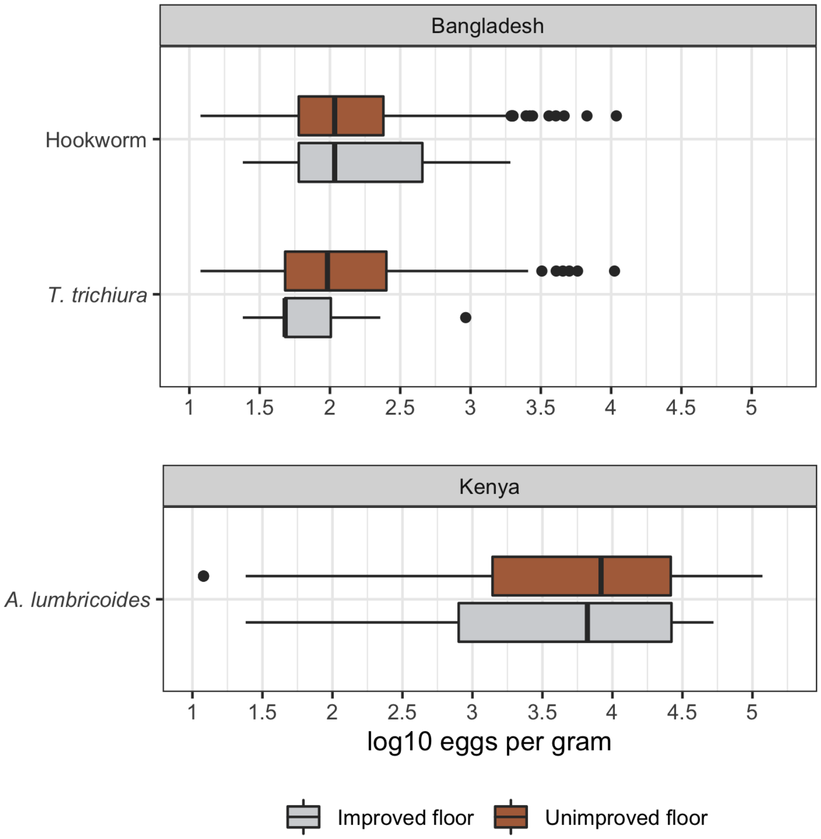

Appendix Figure 3. Adjusted prevalence ratios for household flooring status at enrollment and soil-transmitted helminth and Giardia prevalence at two-year follow-up stratified by potential effect modifiers

A) Bangladesh

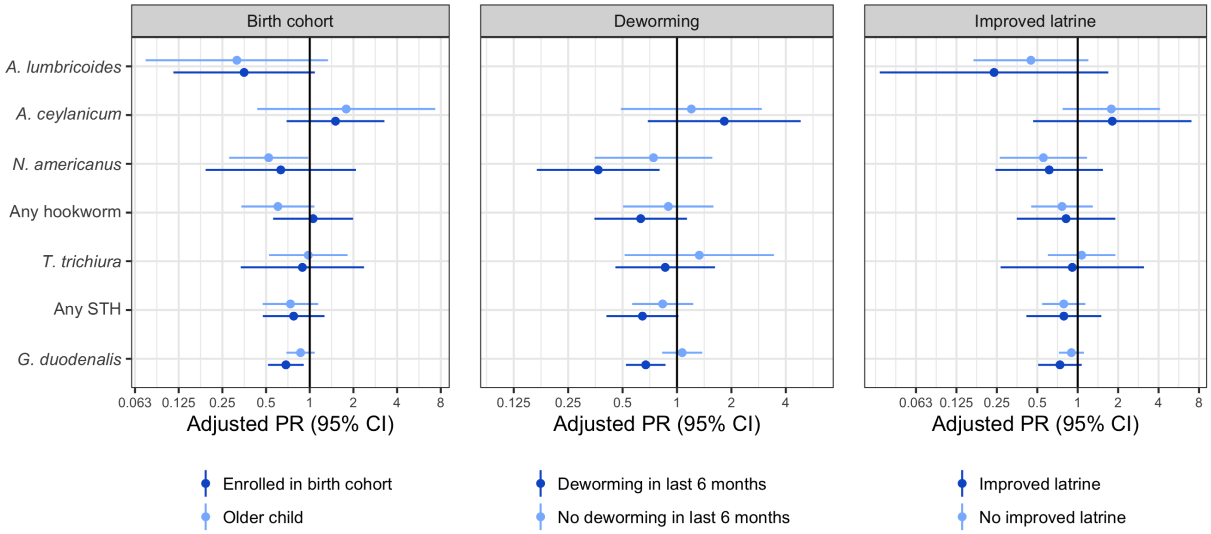

B) Kenya

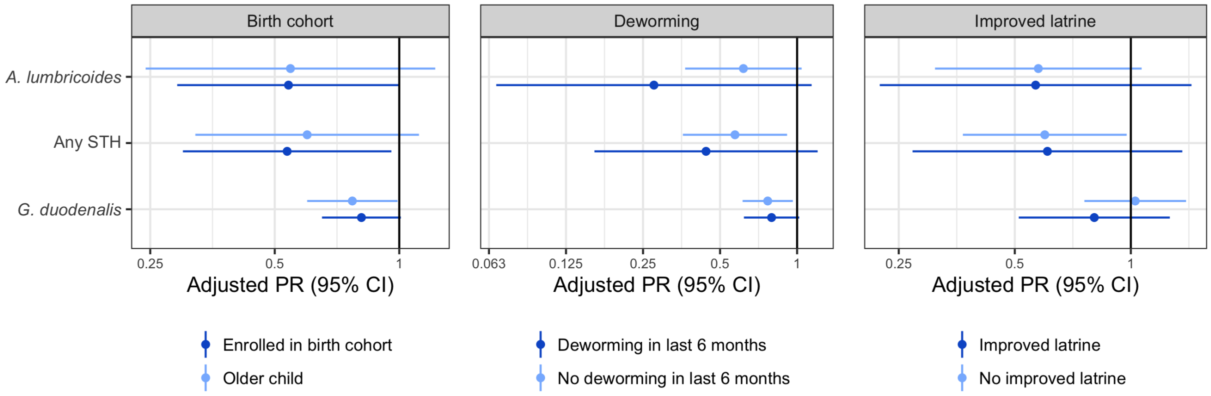

Finished floors includes wood, cement, or tile household floors; unfinished floors were made of soil or earth. In the plots error bars indicate 95% confidence intervals. Prevalence ratios (PRs) compared the prevalence of infection at two-year follow-up in children with improved household flooring at enrolment to those with unimproved household flooring at enrollment. Adjusted PRs control for potential confounders associated with the outcome (see list in the Methods section). Confidence intervals were adjusted for clustering at the village level. Results are not shown for *A. lumbricoides* in Panel A) for deworming because of sparse data. All interaction p-values were ≥ 0.05 except for the p-value for *G. duodenalis* in Bangladesh for child age.

Appendix Figure 4. Predicted probability of household finished floor status

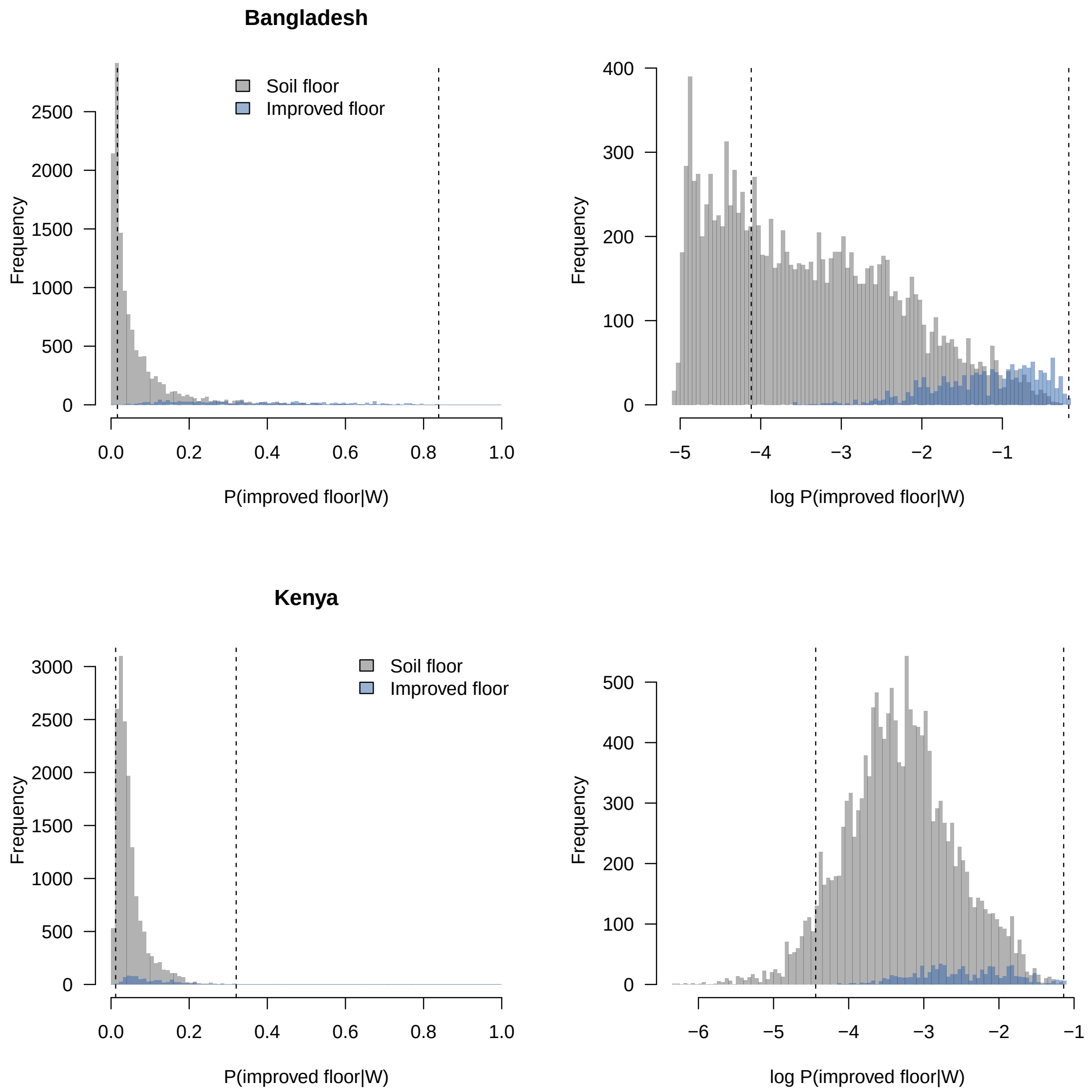

The predicted probability of having a finished floor adjusting for covariates defined above was estimated using an ensemble machine learning algorithm and the following covariates if they were associated with the outcome using a likelihood ratio test (p-value <0.2): month, child age, child sex, birth order (Bangladesh only), mother's age, mother's education, mother's height, number of household members $\leq$ 18 years, total number of individuals in the compound, food insecurity, household assets, and intervention arm.

Appendix Figure 5. Sensitivity analysis accounting for changes in household flooring status during the follow-up period

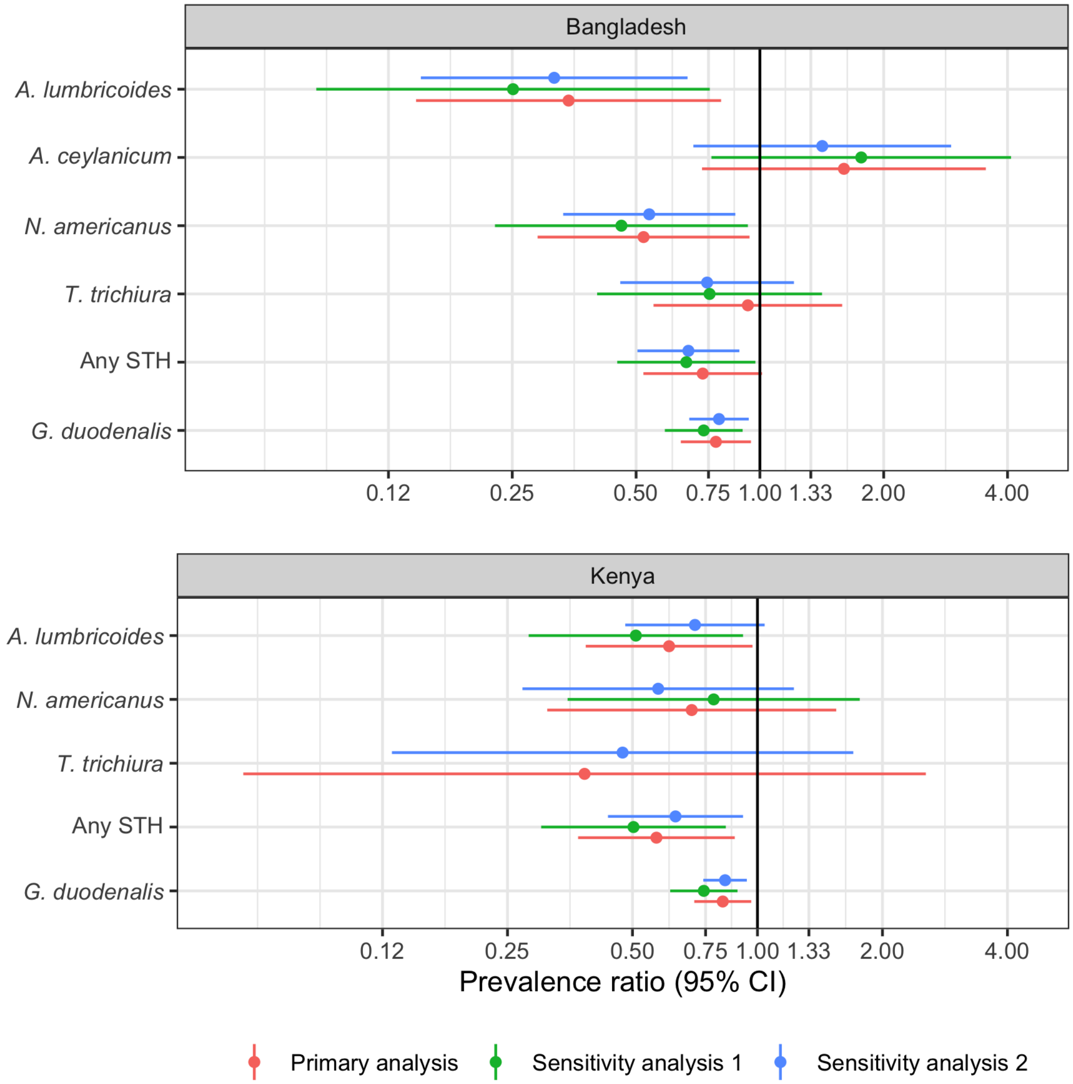

Finished floors includes wood, cement, or tile household floors; unfinished floors were made of soil or earth. In the plots error bars indicate 95% confidence intervals. Prevalence ratios (PRs) compared the prevalence of infection at two-year follow-up in children with improved household flooring at enrolment to those with unimproved household flooring at enrollment. Adjusted PRs control for potential confounders associated with the outcome (see list in the Methods section). Confidence intervals were adjusted for clustering at the village level. Results are not shown for *T. trichiura* in Kenya for Sensitivity Analysis 1 because of sparse data.

Primary analysis: Uses household flooring status at enrollment regardless of changes at two-year follow-up.

Sensitivity analysis 1: Excludes households that changed flooring status between enrollment and two-year follow-up.

Sensitivity analysis 2: Includes households that improved their household flooring status between enrollment and follow-up in the exposure group.

Appendix Table 1. Soil-transmitted helminth and *Giardia duodenalis* prevalence and median Cq value detected by qPCR at two-year follow-up by household flooring status at enrollment

	Finished floors				Unfinished floors			
	N	Prevalence (95% CI)	N	Median Cq value in positive samples (range)	N	Prevalence (95%CI)	N	Median Cq value in positive samples (range)
**Bangladesh**								
A. lumbricoides	255	2.0 (0.4, 3.7)	5	24.8 (22.3, 31.2)	2,544	13.8 (12.0, 15.5)	350	23.3 (16.4, 36.1)
A. ceylanicum	255	4.3 (1.6, 7.4)	11	24.1 (16.5, 37.8)	2,544	3.7 (2.9, 4.6)	95	23.5 (14.4, 37.1)
N. americanus	255	5.1 (2.5, 8.2)	13	20.2 (13.9, 27.2)	2,544	19.7 (17.7, 21.9)	502	20.8 (14.0, 35.5)
T. trichiura	255	5.5 (2.9, 8.6)	14	27.7 (25.2, 36.8)	2,544	12.9 (11.0, 14.9)	329	27.7 (21.4, 40.0)
Any STH	255	14.5 (9.8, 19.7)	–	–	2,544	36.7 (34.1, 39.4)	–	–
G. duodenalis	661	19.4 (16.1, 22.8)	–	–	6,233	33.2 (31.7, 34.6)	–	–
**Kenya**								
A. lumbricoides	174	11.5 (7.0, 17.5)	20	26.2 (18.5, 33.7)	2,924	23.7 (21.3, 25.9)	692	24.8 (16.0, 39.6)
N. americanus	174	3.4 (1.1, 6.5)	6	28.8 (18.7, 34.8)	2,923	7.2 (5.7, 8.8)	209	24.5 (11.1, 39.2)
T. trichiura	174	1.1 (0.0, 3.1)	2	30.0 (27.3, 32.7)	2,922	1.2 (0.6, 2.0)	35	28.5 (23.1, 34.6)
Any STH	173	14.5 (9.3, 20.4)	–	–	2,920	29.2 (26.6, 31.8)	–	–
G. duodenalis	456	31.4 (27.0, 35.9)	–	–	8,436	39.1 (37.8, 40.4)	–	–

This table excludes observations with propensity scores in the highest and lowest 1% of the observed distribution of propensity scores in each country. Propensity scores estimated the probability a child lived in a household with a finished floor conditioning on the following covariates if they were associated with the outcome using a likelihood ratio test (p-value <0.2): month, child age, child sex, birth order (Bangladesh only), mother's age, mother's education, mother's height, number of household members $\leq$ 18 years, total number of individuals in the compound, food insecurity, household assets, and intervention arm. In Kenya, *G. duodenalis* was measured by ELISA, not qPCR. Quantitative estimates of infection intensity were not available for this measure using ELISA.

Appendix Table 2: Soil-transmitted helminth prevalence and infection intensity detected by Kato-Katz at two-year follow-up by household flooring status at enrollment

	Finished floors				Unfinished floors			
	N	Prevalence (95% CI)	N	Geometric Mean EPG in positive samples (95% CI)	N	Prevalence (95% CI)	N	Geometric Mean EPG in positive samples (95% CI)
**Bangladesh**								
Hookworm	691	2.2 (1.2, 3.3)	691	0.12 (0.06, 0.18)	6,496	8.3 (7.4, 9.1)	6,496	0.49 (0.43, 0.56)
T. trichiura	691	2.3 (1.3, 3.6)	691	0.10 (0.05, 0.16)	6,496	7.5 (6.6, 8.4)	6,496	0.43 (0.36, 0.51)
**Kenya**								
A. lumbricoides	471	11.7 (8.5, 14.9)	471	1.62 (0.85, 2.70)	8,568	20.4 (19.1, 21.7)	8,568	4.77 (3.67, 6.12)
Any STH	471	12.1 (8.9, 15.3)	–	–	8,568	22.3 (20.9, 23.6)	–	–

In Bangladesh, results for *A. lumbricoides* are not shown due to concerns about potential misclassification. In Kenya, overall prevalence of any STH in each country including hookworm and *T. trichiura*.

Appendix Table 3: Unadjusted and adjusted prevalence ratios for soil-transmitted helminth infection detected by Kato-Katz

	N	Unadjusted PR (95% CI)	Adjusted PR (95% CI)
**Bangladesh**			
Hookworm	7,187	0.26 (0.16, 0.43)	0.65 (0.39, 1.08)
T. trichiura	7,187	0.31 (0.19, 0.51)	0.57 (0.33, 0.96)
**Kenya**			
A. lumbricoides	9,039	0.57 (0.43, 0.76)	0.69 (0.51, 0.93)
Any STH	9,039	0.54 (0.42, 0.71)	0.66 (0.50, 0.88)

Prevalence ratios (PRs) compared the prevalence of infection at two-year follow-up in children with improved household flooring at enrolment to those with unimproved household flooring at enrollment. Adjusted PRs control for potential confounders associated with the outcome (see list in the Methods section). Confidence intervals were adjusted for clustering at the village level. In Bangladesh, results for *A. lumbricoides* are not shown due to concerns about potential misclassification.

Appendix Table 4: Unadjusted and adjusted soil-transmitted helminth fecal egg count differences detected by Kato-Katz among infected individuals at two-year follow-up by household flooring status at enrollment

	N	Unadjusted FECD (95% CI)	Adjusted FECD (95% CI)
**Bangladesh**			
Hookworm	554	0.37 (-0.63, 1.38)	0.49 (-2.40, 3.38)
T. trichiura	502	-0.60 (-0.93, -0.28)	-0.45 (-0.59, -0.32)
**Kenya**			
A. lumbricoides	1,802	-0.05 (-0.39, 0.29)	0.06 (-0.05, 0.17)

Fecal egg count differences (FECDs) are defined as the ratio of mean eggs per gram among positive samples detected by Kato-Katz minus one. Adjusted estimates control for potential confounders associated with the outcome (see list in the Methods section). Confidence intervals were adjusted for clustering at the village level. In Bangladesh, results for *A. lumbricoides* are not shown due to concerns about potential misclassification.

Appendix Table 5: Unadjusted and adjusted prevalence ratios for infection detected by qPCR or ELISA estimated using targeted maximum likelihood estimation

	N	Unadjusted PR (95% CI)	Adjusted PR (95% CI)
**Bangladesh**			
A. lumbricoides	2,796	0.14 (0.07, 0.28)	0.17 (0.10, 0.30)
A. ceylanicum	2,796	1.17 (0.53, 2.58)	1.97 (0.85, 4.57)
N. americanus	2,785	0.26 (0.16, 0.43)	0.53 (0.40, 0.71)
T. trichiura	2,796	0.45 (0.28, 0.71)	1.11 (0.87, 1.43)
Any STH	2,796	0.40 (0.29, 0.55)	0.71 (0.58, 0.86)
G. duodenalis	6,874	0.58 (0.47, 0.71)	0.82 (0.70, 0.95)
**Kenya**			
A. lumbricoides	3,059	0.51 (0.27, 0.97)	0.55 (0.30, 1.03)
N. americanus	2,961	0.53 (0.18, 1.55)	0.51 (0.15, 1.75)
T. trichiura	2,983	0.57 (0.03, 11.61)	0.55 (0.03, 10.28)
Any STH	2,836	0.47 (0.26, 0.84)	0.52 (0.31, 0.89)
G. duodenalis	8,457	0.77 (0.65, 0.92)	0.80 (0.66, 0.96)

Prevalence ratios (PRs) compared the prevalence of infection at two-year follow-up in children with improved household flooring at enrolment to those with unimproved household flooring at enrollment. Adjusted PRs control for potential confounders associated with the outcome (see list in the Methods section). Confidence intervals were adjusted for clustering at the village level.

Appendix Table 6: Unadjusted and adjusted Cq relative difference among infected individuals at two-year follow-up by household flooring status at enrollment estimated using TMLE

	N	Unadj. Cq Relative Difference (95% CI)	Adj. Cq Relative Difference (95% CI)
**Bangladesh**			
A. lumbricoides	355	0.07 (-0.05, 0.18)	0.10 (0.06, 0.14)
A. ceylanicum	106	-0.00 (-0.19, 0.18)	0.06 (-0.12, 0.24)
N. americanus	515	-0.04 (-0.12, 0.04)	-0.04 (-0.11, 0.04)
T. trichiura	341	0.00 (-0.12, 0.12)	0.01 (-0.09, 0.10)
G. duodenalis	2,195	0.01 (-0.02, 0.04)	-0.00 (-0.03, 0.02)
**Kenya**			
A. lumbricoides	712	-0.01 (-0.13, 0.10)	-0.01 (-0.11, 0.10)
N. americanus	206	0.11 (-0.20, 0.41)	0.16 (-0.09, 0.41)
T. trichiura	34	0.01 (-0.15, 0.18)	0.04 (-0.09, 0.17)

Cq ratios compared the arithmetic mean Cq value at two-year follow-up in children with improved household flooring at enrolment to those with unimproved household flooring at enrollment. Adjusted Cq ratios control for potential confounders associated with the outcome (see list in the Methods section). Confidence intervals were adjusted for clustering at the village level

Appendix Table 7. Characteristics at enrollment by household finished flooring status excluding observations with extreme propensity score values

	Bangladesh				Kenya			
	Finished floors		Unfinished floors		Finished floors		Unfinished floors	
	N	Mean / %	N	Mean / %	N	Mean / %	N	Mean / %
**Maternal characteristics**								
Mother's age, years	668	24.2	2122	23.7	464	28.6	8070	27.0
Mother's height, cm	666	151.9	2125	151.5	432	161.8	7779	160.3
At least some primary education	670	13.3	2146	15.7	468	51.3	8138	77.7
At least some secondary education	670	82.8	2146	80.2	468	48.7	8138	22.3
**Compound characteristics**								
# individuals living in compound <=18 yrs	670	1.7	2146	1.7	468	3.5	8136	3.1
Total individuals living in compound	670	9.9	2146	10.7	468	7.4	8138	8.0
**Household characteristics**								
Food secure	670	91.5	2146	89.2	468	96.4	8138	91.3
Has electricity	670	89.4	2146	83.7	468	28.8	8138	6.7
Has improved wall materials	670	91.9	2146	66.5	468	70.3	8138	0.8
Has improved roof material	670	100.0	2146	99.9	468	97.6	8138	66.5
Owns >=1 tv	670	70.7	2146	56.4	468	41.0	8138	11.2
Owns >=1 bicycle	670	35.4	2146	41.7	468	64.3	8138	54.5
Owns >=1 motorcycle	670	23.0	2146	11.2	468	24.6	8138	8.6
Owns >=1 mobile phone	670	98.5	2146	95.2	468	94.7	8138	81.2
**Child characteristics**								
Child age, years	670	4.7	2146	4.7	446	3.7	7846	3.6
Male, %	670	49.3	2146	49.1	468	50.9	8138	47.4

This table excludes observations with propensity scores in the highest and lowest 1% of the observed distribution of propensity scores in each country. Propensity scores estimated the probability a child lived in a household with a finished floor conditioning on the following covariates if they were associated with the outcome using a likelihood ratio test (p-value <0.2): month, child age, child sex, birth order (Bangladesh only), mother's age, mother's education, mother's height, number of household members $\leq$ 18 years, total number of individuals in the compound, food insecurity, household assets, and intervention arm.

Appendix Table 8: Sensitivity analysis excluding observations with extreme predicted probabilities of having finished flooring

	Primary analysis			Sensitivity analysis		
	N	Unadjusted PR (95% CI)	Adjusted PR (95% CI)	N	Unadjusted PR (95% CI)	Adjusted PR (95% CI)
**Bangladesh**						
A. lumbricoides	2,799	0.14 (0.06, 0.34)	0.34 (0.15, 0.80)	1,960	0.19 (0.08, 0.46)	0.35 (0.15, 0.83)
A. ceylanicum	2,799	1.16 (0.55, 2.42)	1.60 (0.72, 3.54)	1,960	1.26 (0.58, 2.75)	1.39 (0.63, 3.08)
N. americanus	2,799	0.26 (0.14, 0.46)	0.52 (0.29, 0.94)	1,960	0.31 (0.17, 0.56)	0.55 (0.30, 1.00)
T. trichiura	2,799	0.42 (0.25, 0.72)	0.93 (0.55, 1.59)	1,960	0.52 (0.31, 0.88)	0.93 (0.54, 1.61)
Any STH	2,799	0.40 (0.28, 0.56)	0.73 (0.52, 1.01)	1,960	0.48 (0.34, 0.68)	0.78 (0.55, 1.09)
G. duodenalis	6,894	0.58 (0.49, 0.70)	0.78 (0.64, 0.95)	4,811	0.65 (0.54, 0.78)	0.80 (0.66, 0.98)
**Kenya**						
A. lumbricoides	3,098	0.49 (0.31, 0.76)	0.61 (0.39, 0.97)	2,936	0.50 (0.31, 0.78)	0.61 (0.38, 0.97)
N. americanus	3,097	0.48 (0.21, 1.08)	0.69 (0.31, 1.55)	2,935	0.48 (0.21, 1.08)	0.70 (0.32, 1.56)
T. trichiura	3,096	0.96 (0.24, 3.88)	0.38 (0.06, 2.54)	2,934	0.91 (0.22, 3.66)	0.47 (0.07, 3.22)
Any STH	3,093	0.49 (0.34, 0.73)	0.57 (0.37, 0.88)	2,931	0.50 (0.34, 0.74)	0.58 (0.39, 0.87)
G. duodenalis	8,892	0.80 (0.70, 0.92)	0.82 (0.70, 0.97)	8,433	0.81 (0.71, 0.94)	0.84 (0.73, 0.97)

Prevalence ratios (PRs) compared the prevalence of infection at two-year follow-up in children with improved household flooring at enrolment to those with unimproved household flooring at enrollment. Adjusted PRs control for potential confounders associated with the outcome (see list in the Methods section). Confidence intervals were adjusted for clustering at the village level. Analyses excluded observations with propensity scores in the highest and lowest 1% of the observed distribution of propensity scores in each country. Propensity scores estimated the probability a child lived in a household with a finished floor conditioning on the following covariates if they were associated with the outcome using a likelihood ratio test (p-value <0.2): month, child age, child sex, birth order (Bangladesh only), mother's age, mother's education, mother's height, number of household members ≤ 18 years, total number of individuals in the compound, food insecurity, household assets, and intervention arm.

Appendix Table 9. E-values for adjusted prevalence ratios

Outcome	Diagnostic	E-value	E-value CI
**Bangladesh**			
Hookworm	Kato-Katz	2.43	1.00
T. trichiura	Kato-Katz	2.93	1.25
A. ceylanicum	qPCR	2.58	1.00
A. lumbricoides	qPCR	5.28	1.79
N. americanus	qPCR	3.24	1.31
T. trichiura	qPCR	1.34	1.00
Any STH	qPCR	2.10	1.00
G. duodenalis	qPCR	1.88	1.28
**Kenya**			
A. lumbricoides	Kato-Katz	2.27	1.35
Any STH	Kato-Katz	2.40	1.53
A. lumbricoides	qPCR	2.65	1.20
N. americanus	qPCR	2.24	1.00
T. trichiura	qPCR	4.66	1.00
Any STH	qPCR	2.90	1.52
G. duodenalis	qPCR	1.72	1.23

E-values quantify the minimum prevalence ratio that an unmeasured confounder would be required to have both household flooring status and each outcome in order for the unmeasured confounder to be completely responsible for a given prevalence ratio. The E-value confidence interval (CI) was calculated for the 95% confidence interval bound closest to the null. E-value CIs were set equal to 1.00 for PR < 1 with upper bounds ≥ 1 and for PR > 1 with upper bounds ≤ 1 (Vanderweele et al., 2017). Large E-values indicate that an unmeasured confounder would have to have strong associations with both household flooring and an outcome in order to explain away our findings; thus, small E-values are indicate that unmeasured confounding may be a larger concern for a given estimate.

STROBE Statement—Checklist of items that should be included in reports of ***cohort studies***

	Item No	Recommendation	Reported in section/sub-section
**Title and abstract**	1	(*a*) Indicate the study's design with a commonly used term in the title or the abstract	Title
		(*b*) Provide in the abstract an informative and balanced summary of what was done and what was found	Abstract
**Introduction**			
Background/rationale	2	Explain the scientific background and rationale for the investigation being reported	Introduction
Objectives	3	State specific objectives, including any prespecified hypotheses	Introduction
**Methods**			
Study design	4	Present key elements of study design early in the paper	Methods/Study design
Setting	5	Describe the setting, locations, and relevant dates, including periods of recruitment, exposure, follow-up, and data collection	Methods/Study design, participants
Participants	6	(*a*) Give the eligibility criteria, and the sources and methods of selection of participants. Describe methods of follow-up	Methods/Participants
		(*b*) For matched studies, give matching criteria and number of exposed and unexposed	NA
Variables	7	Clearly define all outcomes, exposures, predictors, potential confounders, and effect modifiers. Give diagnostic criteria, if applicable	Methods/Exposure measurement, outcome measurement, Statistical methods
Data sources/ measurement	8*	For each variable of interest, give sources of data and details of methods of assessment (measurement). Describe comparability of assessment methods if there is more than one group	Methods/Exposure measurement, outcome measurement
Bias	9	Describe any efforts to address potential sources of bias	Methods/Statistical methods
Study size	10	Explain how the study size was arrived at	Methods/Study size
Quantitative variables	11	Explain how quantitative variables were handled in the analyses. If applicable, describe which groupings were chosen and why	Methods/Outcome measurement, Statistical methods
Statistical methods	12	(*a*) Describe all statistical methods, including those used to control for confounding	Methods/Statistical methods
		(*b*) Describe any methods used to examine subgroups and interactions	Methods/Statistical methods
		(*c*) Explain how missing data were addressed	NA
		(*d*) If applicable, explain how loss to follow-up was addressed	NA
		(*e*) Describe any sensitivity analyses	Methods/Statistical methods
**Results**			

Participants	13*	(a) Report numbers of individuals at each stage of study—eg numbers potentially eligible, examined for eligibility, confirmed eligible, included in the study, completing follow-up, and analysed	Results; Appendix Figure 1
		(b) Give reasons for non-participation at each stage	Appendix Figure 1
		(c) Consider use of a flow diagram	Appendix Figure 1
Descriptive data	14*	(a) Give characteristics of study participants (eg demographic, clinical, social) and information on exposures and potential confounders	Table 1
		(b) Indicate number of participants with missing data for each variable of interest	NA
		(c) Summarise follow-up time (eg, average and total amount)	Results
Outcome data	15*	Report numbers of outcome events or summary measures over time	Results
Main results	16	(*a*) Give unadjusted estimates and, if applicable, confounder-adjusted estimates and their precision (eg, 95% confidence interval). Make clear which confounders were adjusted for and why they were included	Results; Figure 1; Figure 2
		(*b*) Report category boundaries when continuous variables were categorized	Methods/Outcome measurement
		(*c*) If relevant, consider translating estimates of relative risk into absolute risk for a meaningful time period	NA
Other analyses	17	Report other analyses done—eg analyses of subgroups and interactions, and sensitivity analyses	Results
**Discussion**			
Key results	18	Summarise key results with reference to study objectives	Discussion
Limitations	19	Discuss limitations of the study, taking into account sources of potential bias or imprecision. Discuss both direction and magnitude of any potential bias	Discussion
Interpretation	20	Give a cautious overall interpretation of results considering objectives, limitations, multiplicity of analyses, results from similar studies, and other relevant evidence	Discussion
Generalisability	21	Discuss the generalisability (external validity) of the study results	Discussion
**Other information**			
Funding	22	Give the source of funding and the role of the funders for the present study and, if applicable, for the original study on which the present article is based	Abstract; Role of the Funding Source

*Give information separately for exposed and unexposed groups.

Note: An Explanation and Elaboration article discusses each checklist item and gives methodological background and published examples of transparent reporting. The STROBE checklist is best used in conjunction with this article (freely available on the Web sites of PLoS Medicine at http://www.plosmedicine.org/, Annals of Internal Medicine at http://www.annals.org/, and Epidemiology at http://www.epidem.com/). Information on the STROBE Initiative is available at http://www.strobe-statement.org.
